# Tache mongoloïde extensive: un signe clinique qui mérite une attention particulière

**DOI:** 10.11604/pamj.2013.16.41.3062

**Published:** 2013-10-06

**Authors:** Rachid Abilkassem, Aomar Agadr

**Affiliations:** 1 Service de Pédiatrie, Hôpital Militaire d'Instruction Mohamed V, Université Med V, Souissi, Maroc

**Keywords:** Taches mongoloïdes, taches pigmentaires congénitales, gangliosidose, Mongolian spot, congenital spots, gangliosidosis

## Image en médecine

Les taches mongoloïdes sont des taches pigmentaires congénitales, d'un bleu gris, qui traduisent une accumulation de mélanocytes dopa-positifs. Elles sont très fréquentes chez les enfants de couleur. Elles sont habituellement ronde ou ovoïde, peuvent être unique ou multiple, classiquement elles sont bénigne et limité, elles apparaissent à la naissance ou dans les premiers mois de vie et disparaissent spontanément avant la puberté, le siège de prédilection est représenté par la région sacro coccygien. Toutefois lorsqu'elles sont extensives, il mérite une attention particulière. Une association entre taches mongoloïdes et erreur innée du métabolisme notamment la gangliosidose à GM1 et la mucopolyssacharidose a été décrit. Nous rapportons l'observation d'un nourrisson âgé de 15 mois, né à terme, avec une bonne adaptation à la vie extra-utérine. Le développement psychomoteur a été normal jusqu’à l’âge de 6 mois. Elle a été hospitalisée pour exploration d'une encéphalopathie progressive. Le diagnostic de gangliosidose à GM 1 a été retenu sur les éléments suivants:un faciès pseudo hurlerien, une hypotonie axiale, une microcéphalie, des réflexes osteotendineux diminués, un retard psychomoteur important, un strabisme convergent, un nystagmus de l'oeil droit et une atrophie chorio-rétiniènne au fond d'oeil, une énorme tache mongoloïde et une atrophie cortico-souscorticalesur l'imagerie cérébrale. Le dosage de la bétagalactosidase dans le sérum, les leucocytes a montré activité nulle confirmant le diagnostic de gangliosidose à GM1. L'association d'une tache mongoloïde et d'une encéphalopathie progressive doit vous faire évoquer le diagnostic de gangliosidose à GM 1 dans un contexte clinique compatible.

**Figure 1:**
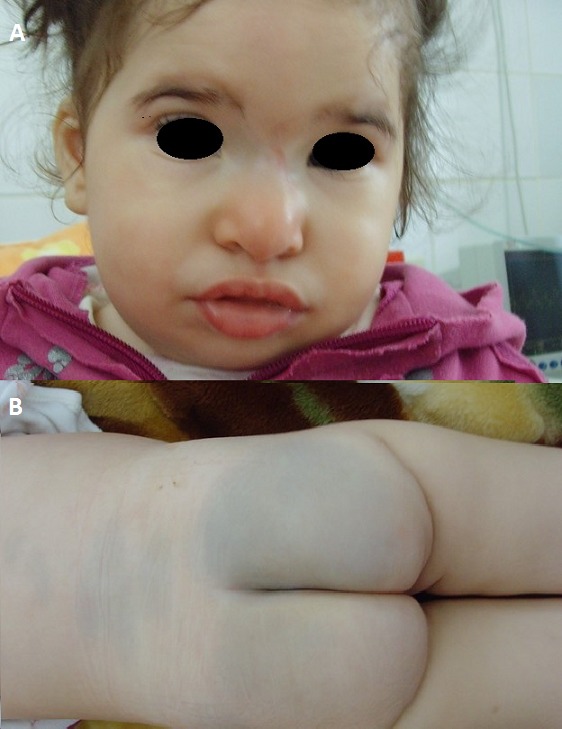
A) Faciès pseudohurlerien; B) Enorme tache mongoloïde

